# Correlation between acute cellular rejection detected with cryobiopsy and elevated donor-derived cell-free DNA in lung transplant recipients

**DOI:** 10.1016/j.jhlto.2025.100463

**Published:** 2025-12-04

**Authors:** Carolin Steinack, Macé M. Schuurmans, Silvan M. Vesenbeckh, René Hage, Zsofia Rosselli, Silvia Ulrich, Malcolm Kohler, Jan Rüschoff, Martina Haberecker, Maurice Roeder, Thomas Gaisl

**Affiliations:** a Department of Pulmonology, University Hospital Zurich, Zurich, Switzerland; b Department of Pathology and Molecular Pathology, University Hospital Zurich, Zurich, Switzerland; c Faculty of Medicine, University of Zurich, Zurich, Switzerland

**Keywords:** Acute cellular rejection, Cryobiopsy, Cell-free DNA, Lung transplant, Transbronchial biopsy

## Abstract

**Introduction:**

Donor-derived cell-free DNA (dd-cfDNA) may be a promising biomarker for detecting acute cellular rejection (ACR) in lung transplant recipients (LTR) without the need for invasive transbronchial biopsies. We aimed to validate a clinical plasma dd-cfDNA assay for the detection of ACR, as determined by cryobiopsy, and to assess its clinical utility.

**Methods:**

In this prospective cohort, dd-cfDNA fraction was measured using a novel single-nucleotide polymorphism-based assay in LTR undergoing surveillance bronchoscopy with cryobiopsies 2, 4, 6, and 12 months after transplantation (and when indicated). Performance characteristics were calculated for LTR without ACR and LTR with ACR (defined as ACR based on pathological assessment of the cryobiopsies ≥A1).

**Results:**

The incidence of ACR (A1 (*N* = 2), grade A2 (*N* = 3), grade A3 (*N* = 1), and no grade A4 or antibody-mediated rejection) was 14% in 43 samples of 39 LTR. The median dd-cfDNA fraction was similar for the stable cohort and the cohort with ACR (median 0.41% [0.15% to 0.72%] vs 0.56% [0.10% to 3.07%], *p* = 0.630). The area under the receiver operator characteristic curve for ACR was 59.3% (95% CI 38.3%−80.3%). Using a ≥1% dd-cfDNA fraction threshold (≥0.5% for single lung transplantations), the negative predictive value for ACR was 87.9% (95% CI 74.1%-97.6%), and the positive predictive value was 20.0% (95% CI 8.0%-32.0%). In the sensitivity analysis, altering the ACR category (≥A1 vs ≥A2) or the dd-cfDNA threshold >0.85% did not produce significant changes in the outcomes.

**Conclusion:**

The incidence of ACR (≥A1 or ≥A2) did not appear to be closely associated with the fraction of dd-cfDNA. More research with a larger sample size and long-term follow-up is needed to evaluate the association between dd-cfDNA and ACR incidence detected by cryobiopsy.

## Background

Despite advances in post-transplant care, current strategies for monitoring lung transplant recipients (LTR) remain suboptimal. Nearly 50% of LTR develop chronic lung allograft dysfunction (CLAD) within 5 years of lung transplantation (LT), often preceded by episodes of acute cellular rejection (ACR), a key risk factor for CLAD progression.^1^, ^2^ One commonly used method to detect ACR in asymptomatic LTR is surveillance bronchoscopy, during which tissue samples from the transplanted lung are routinely obtained. These procedures aim to identify subclinical rejection, guide immunosuppressive therapy, and potentially delay CLAD onset.

Traditionally, forceps biopsies have been used for surveillance tissue sampling; however, transbronchial cryobiopsies (CB) have emerged as a promising alternative. Compared to forceps, CB yields larger, higher-quality specimens with fewer crush and bleeding artifacts, improving histopathologic assessment without increasing the risk of complications such as pneumothorax or severe hemorrhage.^3^, ^4^, ^5^ Nevertheless, an accurate, non-invasive, and cost-effective approach for ACR detection remains an unmet clinical need.^6^

A non-invasive blood-based test detecting donor-derived cell-free DNA (dd-cfDNA) may be a promising biomarker for detecting cell injuries such as ACR, antibody-mediated rejection, CLAD, and pulmonary graft infection without the need for invasive transbronchial biopsies.^7^ Dd-cfDNA has already been utilized in kidney, heart, and liver transplants for graft monitoring.^8^, ^9^, ^10^ These methods encompass a range of approaches, including whole-genome sequencing, targeted genome sequencing, digital droplet PCR, and quantitative PCR.^11^, ^12^ The latter was applied by screening blood plasma of LTRs for quantitative dd-cf-DNA using a panel of more than 13,000 single-nucleotide polymorphisms (Natera, Inc., Austin, TX).^7^ Different inflammations, such as chronic infection, gastroesophageal reflux, or antibody-mediated rejection, can trigger the release of dd-cfDNA.^13^, ^14^ Hence, dd-cfDNA identifies acute rejection and other clinical complications that may be missed by histopathology obtained by forceps biopsy, reinforcing its value as a noninvasive marker of allograft injury.^13^ In recent years, data on LTR have been published, ranging from an early allograft injury marker associated with primary graft dysfunction in the first 7 days posttransplant to patients with CLAD more than 5 years after LT.^13^, ^14^, ^15^, ^16^, ^17^, ^18^, ^19^, ^20^, ^21^

However, no studies to date have compared dd-cfDNA levels with histopathological findings from CB in LTRs. This is relevant because evidence from a recent randomized controlled trial has shown that the incidence of ACR is about 5 times higher when using CB compared to conventional forceps biopsies, highlighting the superior diagnostic sensitivity of CB.^5^ This suggests that prior studies relying solely on forceps-obtained tissue may have significantly underestimated accurate ACR rates due to the limited diagnostic yield of smaller, artifact-prone specimens.^4^

We therefore aimed to validate a clinical plasma dd-cfDNA assay for detecting “minimal” ACR (grade A1) or higher, as determined by CB, and to evaluate its potential clinical utility in lung transplant surveillance.

## Materials and methods

### Patient selection and overall study design

Between July 2023 and December 2023, all adult LTRs aged 18 years and older undergoing clinically indicated and routinely performed bronchoscopy with CB 2, 4, 6, and 12 months after LT at the University Hospital Zurich were included in this investigator-initiated, prospective single-center study. Patients were excluded if they had any contraindications for bronchoscopy (e.g., co-morbidities): international normalized ratio (INR) > 2, thrombocytes <50,000 per microliter of blood, double antiplatelet drugs (e.g., aspirin and clopidogrel) within 7 days before bronchoscopy, oral anticoagulants with non-vitamin K antagonist within 48 hours before biopsy, or relevant pulmonary hypertension (mean pulmonary arterial pressure > 30 mmHg or estimated gradient between the right ventricle and the right atrium > 35 mmHg). LTRs with pulmonary infection or neutropenia were also excluded from the study. Pathologists dedicated to LT evaluations assessed all biopsies according to ISHLT criteria (A0-A4).^22^ In cases of ACR grade A1, the prednisolone dosage was increased by 10 mg/day for 5 days. In cases of grade A2 rejection, intravenous steroid pulse therapy with methylprednisolone 125 mg/day (1-2 mg/kg/day) for 3 days was given. In cases of persistent grade A2 or A3 rejection, the methylprednisolone pulse dose (10-15 mg/kg/day) was increased to 0.5 or 1 g/day over 3 days, and the calcineurin inhibitor was switched if tacrolimus was not yet administered. All patient-related data, including clinical, demographic, and pathological information, as well as reports from bronchoscopy and spirometry, were obtained from electronic patient records. The ethics committee of the Canton of Zurich approved the study (BASEC-ID 2021-00466), which was registered at clinicaltrials.gov (NCT05006742). The study was consistent with the ISHLT ethical statement. All participants provided written informed consent.

### Bronchoscopy and dd-cfDNA analysis

Vitamin K-antagonist anticoagulants were discontinued 5 to 7 days before the procedure. Patients with an INR > 1.3 and ≤ 2 received vitamin K, so the INR was ≤ 1.3 on the day of bronchoscopy. Bronchoscopies were usually conducted in moderate sedation using local anesthesia (lidocaine) and intravenous propofol. Flexible bronchoscopes (Olympus, Tokyo, Japan, 190 series) were inserted through an uncuffed (BronchoFlex, Rüsch, size inner diameter 7.5 mm, Germany) or cuffed tracheal tube (size 7.5 to 8.5) to prevent laryngeal injury and to maintain a safe airway in case of complications. All bronchoscopies were performed as recently described.^5^ Until today, forceps biopsies are the gold standard to detect ACR in patients following LT. CB are not routinely used to assess ACR in LTRs.^1^, ^2^ For histological assessment, 2 CB from 2 different lobes were obtained. We adhered to our protocol based on recently published data^4^, ^5^ Pneumothorax was screened with chest radiography in an upright position 2-4 hours after the procedure. The bleeding grade was scored according to the Nashville Bleeding Score.^23^

Peripheral blood samples were collected before each fiberoptic bronchoscopy in 10-mL Streck tubes. Dd-cfDNA fraction was measured using a novel single-nucleotide polymorphism-based assay. Plasma was isolated through 2 centrifugation steps and stored at −80 °C in deidentified 15-mL Falcon tubes. Samples were batch analyzed using the Prospera test (Natera, Inc.) at Natera’s CLIA-certified, CAP-accredited lab in San Carlos, CA.^7^

### Data analysis

Performance characteristics were calculated for LTR without ACR (stable cohort) and LTR with ACR (defined as signs of ACR based on pathological assessment of the CB ≥A1, regardless of grade B). The primary outcome was to validate a clinical plasma dd-cfDNA assay for ACR detection, as adjudicated by CB according to the ISHLT histopathological criteria, and to explore its clinical value.^22^

Continuous outcomes were summarized using the mean ± SD or median (interquartile range) as appropriate. The prespecified analyses and safety analysis used data from the full-analysis set, which included all patients randomly assigned to a study group and had at least 1 CB.^5^ No missing values were imputed. Prespecified intraindividual diagnostic analyses included participants with ACR and those without ACR.

The primary outcome was analyzed using multilevel mixed-effects logistic regression without weights to compare dichotomous variables between groups. Categorical data were compared using Pearson’s χ² or Fisher’s exact test as appropriate, and inference for stratified categorical data. For continuous data, between-group comparisons were performed by Student’s *t*-test for parametric variables or the Mann-Whitney *U* test for non-parametric variables. For statistical significance, a 2-sided *p*-value of less than 0.05 was used. All analyses were conducted using STATA version 18.0 (StataCorp LP, College Station, TX, USA).

## Results

### Baseline characteristics

Between July 14, 2023, and December 18, 2023, 39 out of 41 patients were enrolled. Two patients were excluded because 1 had a pulmonary infection and the other declined participation in the study. A total of 44 procedures (from 39 individuals, as 5 had 2 procedures) were analyzed; in 1 case, the CB was deemed non-diagnostic based on ISHLT criteria, resulting in 43 evaluable CB and corresponding dd-cfDNA levels for analysis, as per the study protocol.

Demographic parameters are shown in Table 1**.** All LTR received lifelong triple immunosuppression, consisting of a corticosteroid, a calcineurin inhibitor (tacrolimus or ciclosporin), and an antimetabolite (mycophenolate mofetil) or an mTOR inhibitor (everolimus). In all patients, 2 CB were obtained from 2 different lobes. The size of the CB had a median of 2 mm (IQR 2-2).

**Table 1:** Baseline Characteristics of the Patients

Lung transplant recipients, *n*	39
Age, year ± SD	57.2 ± 9.7
Sex - *n* (%)	
Female	9 (23.1%)
Male	30 (76.9%)
Disease - *n* (%)	
COPD	29 (74.4%)
Sarcoidosis	1 (2.6%)
Interstitial lung disease	6 (15.4%)
Cystic fibrosis	1 (2.6%)
Sjögren syndrome	1 (2.6%)
Bronchiolitis	1 (2.6%)
Side of lung transplantation - *n* (%)	
Bilateral	33 (84.6%)
Unilateral	6 (15.4%)

Plus-minus values are mean ± standard deviation. Values in square brackets are median [interquartile range]. Air trapping is detected on chest computed tomography by the depiction of hyperinflated lung areas due to abnormal air retention and may be observed in patients with chronic lung allograft dysfunction. COPD denotes chronic obstructive pulmonary disease, INR international normalized ratio, FEV1 forced expiratory volume in the first second, and DLCO diffusing capacity for carbon monoxide.

### Outcomes

The incidence of ACR (A1 (*N* = 2), grade A2 (*N* = 3), grade A3 (*N* = 1), and no grade A4 or antibody-mediated rejection) was 14% in 43 samples of 39 LTRs (Table 2). The median dd-cfDNA fraction was similar for the stable cohort and the cohort with ACR (median 0.41% [0.15% to 0.72%] vs 0.56% [0.10% to 3.07%], *p* = 0.630).

**Table 2:** Diagnostic Parameters in the Entire Per-protocol Analysis (*n* = 43 Procedures From 39 Individuals)

	Non-ACR cohort	ACR cohort	*p*-value
Number of procedures - *n*	37	6	-
Days since lung transplantation	224 [132-377]	197 [120-307]	0.609
Side of biopsy - *n* (%)			-
Right	20 (54.1%)	3 (50.0%)	-
Left	17 (45.9%)	3 (50.0%)	-
Air trapping - *n* (%)	8 (21.7%)	1 (16.7%)	0.379
Grade of ACR - *n* (%)			
A0 A1 A2 A3 A4	37 (100%) 0 (0%) 0 (0%) 0 (0%) 0 (0%)	0 (0%) 2 (33.3%) 3 (50.0%) 1 (16.7%) 0 (0%)	-
Indication bronchoscopy - *n* (%)	7 (18.9%)	1 (16.7%)	0.699
dd-cfDNA fraction	0.41% [0.15% to 0.72%]	0.56% [0.10% to 3.07%]	0.630
Hemoglobin, G/L	111 [100-123]	108 [98-120]	0.568
Leucocytes, G/L	6.2 [4.5-8.6]	5.4 [4.1-8.3]	0.865
Lymphocytes, G/L	0.6 [0.4-1.0]	0.5 [0.4-0.9]	0.684
Thrombocytes, G/L	245 [208-302]	237 [177-266]	0.538
INR	1.0 [0.9-1.0]	1.0 [0.9-1.0]	0.901
C-reactive protein, mg/l	1.0 [0.6-2.3]	0.8 [0.6-2.5]	0.253
FEV1, liters	2.3 [1.8-3.1]	2.3 [1.8-2.9]	0.190
DLCO, %pred.	62 [51-76]	56[49–71]	0.124

Values in square brackets are median [interquartile range]. INR denotes international normalized ratio, FEV1 forced expiratory volume in the first second, and DLCO diffusing capacity for carbon monoxide.

ACR, acute cellular rejection; dd-cfDNA, donor-derived cell-free DNA.

The area under the receiver operator characteristic curve for ACR was 59.3% (95% CI 38.3%-80.3%). Using a ≥1% dd-cfDNA fraction threshold (≥0.5% for single LTs), the negative predictive value for ACR was 87.9% (95% CI 74.1%-97.6%), and the positive predictive value was 20.0% (95% CI 8.0%-32.0%). In the sensitivity analysis, altering the ACR category (≥A1 vs ≥A2) or the dd-cfDNA threshold > 0.85% did not produce significant changes in the outcomes (Table 3). Regression analysis suggested that neither C-reactive protein (*p* = 0.245), presence of donor-specific antibodies (*p* = 0.468), or indication-bronchoscopy (*p* = 0.561) was associated with the incidence of ACR in the entire cohort.

**Table 3:** Sensitivity, Specificity, and Predictive Values for the Entire Cohort (*n* = 43) Using Different dd-cfDNA Fraction Thresholds

	Value (%)	95% Confidence interval
ACR prevalence	14.0	3.6-24.3
*≥1% dd-cfDNA fraction threshold (≥0.5% for single lung transplantations)*		
Sensitivity	33.3	19.2-47.4
Specificity	78.4	66.1-90.7
Positive predictive value	20.0	8.0-31.9
Negative predictive value	87.9	78.1-97.6
*≥0.85% dd-cfDNA fraction threshold (≥0.43% for single lung transplantations)*		
Sensitivity	50.0	35.1-64.9
Specificity	75.7	62.9-88.5
Positive predictive value	25.0	12.1-37.9
Negative predictive value	90.3	81.5-99.2

dd-cfDNA, Donor-derived cell-free DNA.

### Procedure characteristics

No pneumothorax was detected in any of the procedures, as confirmed by post-interventional ultrasound and subsequent chest X-ray performed within 4 hours. Bleeding complications occurred in several cases: Grade 1 in 17.5%, grade 2 in 31.0%, and grade 3 in 7.9% of procedures; no grade 4 bleeding was observed. No deaths occurred during the 7-day post-procedural observation period. Bleeding severity did not correlate with the use of antiplatelet agents (*p* = 0.834) or anticoagulants (*p* = 0.190), which were administered in 7 and 10 patients, respectively. Additionally, there was no significant association between time since LT and ACR grade (*p* = 0.127).

## Discussion

In our study, the 6 cases of ACR (≥A1 or ≥A2) were not clearly linked with the fraction of dd-cfDNA (independent of the dd-cfDNA threshold ≥0.85 or ≥1% for bilateral LT, ≥0.5% for single LT). Furthermore, the fraction of dd-cfDNA did not differ significantly between patients with histologically proven ACR (*n* = 6) and without ACR (*n* = 37). Possible reasons for these findings include the use of CB, which allows for more sensitive detection of minimal ACR, a small sample size, and an overall low incidence of ACR. Especially the latter does not exclude dd-cfDNA as a potential, non-invasive diagnostic tool in patients following LT. Given the limited incidence of ACR, our results are not conclusive when it comes to establishing a clear association between dd-cfDNA and ACR. Further research with long-term follow-up is needed to explore the correlation between CB and dd-cfDNA.

Considering the advantages of obtaining larger, higher-quality specimens with fewer artifacts during shorter fluoroscopy and procedure times without increasing risks of pneumothorax and bleeding makes CB a favorable biopsy option.^24^, ^25^, ^3^ We recently reported that in 23.8% of patients who underwent both consecutive CB and forceps biopsy during the same procedure, ACR would have been missed if only forceps biopsy had been performed.^4^ Even though the optimal number of specimens obtained with the cryoprobe is unclear, with literature suggesting 2 to 3 samples, often from different lobes, our recent research indicates that 2 samples obtained with the 2.4 probe from different lobes appear to be sufficient.^3^, ^4^, ^5^, ^24^, ^26^

Greater variability in dd-cfDNA over time was linked to an increased risk of mortality after heart transplantation.^8^ In recent years, ddcf-DNA has been used in LTR. Overall, dd-cfDNA levels were higher in LTR than in other heart transplant recipients.^17^ An early rise in dd-cfDNA 3 months after LT was associated with a 6.6-fold increase in the risk of allograft failure.^19^ Dd-cfDNA levels were significantly higher after transplant surgery, reaching a low baseline of 0.21% at 4 months and remaining stable until 9 months, then increasing to 0.60% to 0.68% between 18 and 24 months. The median dd-cfDNA fraction for the stable group was 0.41% [0.15% to 0.72%], including patients around 217 days posttransplant.^13^, ^15^ Jang et al found that 16.3% of ACR cases had dd-cfDNA, with a dd-cfDNA level in ACR grade 1 being 1.5 times higher than in grade 0 (0.68% vs 0.41%, *p* = 0.030). In contrast, the dd-cfDNA level in grade ≥ 2 was twice as high as in grade 1. The long-term outcome of ACR Grade A1 warrants further investigation in future long-term studies. As a result, different treatment regimens are applied across various lung transplant centers. Histopathology was only provided in 31% of cases with elevated dd-cfDNA using a threshold of ≥1.0% for ACR, likely due to the smaller tissue samples obtained with forceps biopsy.^13^ We observed 14% ACR, with median dd-cfDNA fractions similar in both the stable cohort and those with ACR (median 0.41% [0.15% to 0.72%] vs 0.56% [0.10% to 3.07%], *p* = 0.630). This may be due to the smaller sample size and the higher sensitivity of CB in detecting ACR.

Nonetheless, evidence on circulating dd-cfDNA in graft rejection after LT remains inconsistent. Dd-cfDNA can be elevated in different types of lung inflammation, such as pulmonary infections.^27^ CRP did not differ between the groups with or without ACR in our cohort.

Our study has several limitations. First, it is based on a single-center analysis, which may limit the generalizability of the findings, particularly for centers with different surveillance programs. Second, a limited sample size, particularly for the LTRs with detected ACR. Furthermore, the incidence of ACR in bronchoscopies performed for clinical indication was only 16.7% (1 out of 8 procedures) and relatively low when compared to our recent research (47%; 8 out of 17 procedures). The likely optimization of post-transplant care is one of the main reasons for the low incidence of ACR. Seven out of 8 bronchoscopies performed for clinical indication showed no histologically proven ACR. Reasons included 2 cases of pulmonary infection, 4 cases of volume overload, and 1 stricture of the right anastomosis, all of which resulted in pulmonary symptoms or lung function decline. Third, the lack of longitudinal data. Our study includes 43 procedures performed in 39 patients, with 5 patients undergoing 2 procedures and 34 undergoing only one, over a 6-month study period. Future studies should incorporate longitudinal dd-cfDNA measurements to interpret dd-cfDNA kinetics. The greatest strength of our trial lies in its being the first prospective trial comparing histological data obtained with CB with a noninvasive blood test detecting dd-cfDNA in lung transplants. Further study will focus on long-term outcomes correlating CB with dd-cfCNA. In conclusion, our findings support the use of CB as the current primary method for histological assessment of ACR in LTRs. Given the limited diagnostic performance of dd-cfDNA observed in our study when benchmarked against the gold standard CB, further investigation is required before recommending its routine use for ACR detection. Larger prospective studies in diverse patient populations are warranted to further explore the potential role of dd-cfDNA in LT monitoring. Additionally, long-term follow-up is necessary to comprehensively evaluate the safety profile of CB in this vulnerable population.

## Financial support

This study was funded by Natera Inc.

## Ethics statement

The study was performed in the Department of Pulmonology at the University Hospital Zurich, Zurich, Switzerland, and approved by the Cantonal Ethics Committee in Zurich, Switzerland (ID-2021-00466).

## CRediT authorship contribution statement

Carolin Steinack and Thomas Gaisl had full access to all data in the study and take responsibility for the integrity of the data and the accuracy of the data analysis. Study concept and design: Carolin Steinack. Acquisition of data: Carolin Steinack, Thomas Gaisl, Maurice Roeder, Silvan M. Vesenbeckh, Jan Rüschoff, Martina Haberec. Analysis or interpretation: Carolin Steinack, Jan Rüschoff, Silvia Ulrich, Martina Haberecker, Zsofia Rosselli, Malcolm Kohler, and Thomas Gaisl. Drafting of the manuscript: Carolin Steinack, Thomas Gaisl. Critical revision of the manuscript for important intellectual content: All authors. Statistical analysis: Thomas Gaisl. Administrative, technical, or material support: Carolin Steinack, Jan Rüschoff, Martina Haberecker, and Thomas Gaisl. Study supervision: Macé M. Schuurmans.

## Conflicts of Interest statement

The authors declare the following financial interests/personal relationships, which may be considered as potential competing interests: M. Kohler received consulting fees from Novartis. He is co-founder and shareholder of Deep Breath Intelligence AG, a company that provides services in the field of breath analysis.

## Declaration of Generative AI and AI-assisted technologies in the writing process

During the preparation of this work, the authors used *ChatGPT* to improve readability. After using this tool, the authors reviewed and edited the content as needed and take full responsibility for the content of the published article.

## Acknowledgments

Natera supported the study by providing dd-cfDNA analyses free of charge. No additional payments or transactions were involved. This was an investigator-initiated trial; Natera had no role in the study design, data analysis, or publication process.

## Data Availability

The data supporting this study's findings are available upon reasonable request from the corresponding author.

## References

[1] Chambers D.C., Yusen R.D., Cherikh W.S. (2017). The Registry of the International Society for Heart and Lung Transplantation: thirty-fourth adult lung and heart-lung transplantation report-2017; Focus theme: allograft ischemic time. J Heart Lung Transpl.

[2] Todd J.L., Neely M.L., Kopetskie H. (2020). Risk factors for acute rejection in the first year after lung transplant. A multicenter study. Am J Respir Crit Care Med.

[3] Gershman E., Ridman E., Fridel L. (2018). Efficacy and safety of trans-bronchial cryo in comparison with forceps biopsy in lung allograft recipients: analysis of 402 procedures. Clin Transpl.

[4] Steinack C., Gaspert A., Gautschi F. (2022). Transbronchial cryobiopsy compared to forceps biopsy for diagnosis of acute cellular rejection in lung transplants: analysis of 63 consecutive procedures. Life.

[5] Steinack C., Roeder M., Vesenbeckh S. (2025). Transbronchial cryobiopsy alone versus combined with traditional forceps biopsy for acute cellular rejection in lung transplant recipients. A diagnostic randomized trial. JHLT Open.

[6] Chhajed P.N., Aboyoun C., Malouf M.A., Hopkins P.M., Plit M.L., Glanville A.R. (2003). Risk factors and management of bleeding associated with transbronchial lung biopsy in lung transplant recipients. J Heart Lung Transpl.

[7] Rosenheck J.P., Ross D.J., Botros M. (2022). Clinical validation of a plasma donor-derived cell-free DNA assay to detect allograft rejection and injury in lung transplant. Transpl Direct.

[8] Kamath M., Shekhtman G., Grogan T. (2022). Variability in donor-derived cell-free DNA scores to predict mortality in heart transplant recipients - a proof-of-concept study. Front Immunol.

[9] Schutz E., Asendorf T., Beck J. (2020). Time-dependent apparent increase in dd-cfDNA percentage in clinically stable patients between one and five years following kidney transplantation. Clin Chem.

[10] Schutz E., Fischer A., Beck J. (2017). Graft-derived cell-free DNA, a noninvasive early rejection and graft damage marker in liver transplantation: a prospective, observational, multicenter cohort study. PLoS Med.

[11] Grskovic M., Hiller D.J., Eubank L.A. (2016). Validation of a clinical-grade assay to measure donor-derived cell-free DNA in solid organ transplant recipients. J Mol Diagn.

[12] Drag M.H., Kilpelainen T.O. (2021). Cell-free DNA and RNA-measurement and applications in clinical diagnostics with focus on metabolic disorders. Phys Genom.

[13] Jang M.K., Tunc I., Berry G.J. (2021). Donor-derived cell-free DNA accurately detects acute rejection in lung transplant patients, a multicenter cohort study. J Heart Lung Transpl.

[14] Levine D.J., Ross D.J., Sako E. (2020). Single center "snapshot" experience with donor-derived cell-free DNA after lung transplantation. Biomark Insights.

[15] Keller M., Bush E., Diamond J.M. (2021). Use of donor-derived-cell-free DNA as a marker of early allograft injury in primary graft dysfunction (PGD) to predict the risk of chronic lung allograft dysfunction (CLAD). J Heart Lung Transpl.

[16] Tanaka S., Sugimoto S., Kurosaki T. (2018). Donor-derived cell-free DNA is associated with acute rejection and decreased oxygenation in primary graft dysfunction after living donor-lobar lung transplantation. Sci Rep.

[17] De Vlaminck I., Martin L., Kertesz M. (2015). Noninvasive monitoring of infection and rejection after lung transplantation. Proc Natl Acad Sci U. S. A..

[18] Zou J., Duffy B., Slade M. (2017). Rapid detection of donor cell free DNA in lung transplant recipients with rejections using donor-recipient HLA mismatch. Hum Immunol.

[19] Agbor-Enoh S., Wang Y., Tunc I. (2019). Donor-derived cell-free DNA predicts allograft failure and mortality after lung transplantation. EBioMedicine.

[20] Agbor-Enoh S., Jackson A.M., Tunc I. (2018). Late manifestation of alloantibody-associated injury and clinical pulmonary antibody-mediated rejection: evidence from cell-free DNA analysis. J Heart Lung Transpl.

[21] Khush K.K., De Vlaminck I., Luikart H., Ross D.J., Nicolls M.R. (2021). Donor-derived, cell-free DNA levels by next-generation targeted sequencing are elevated in allograft rejection after lung transplantation. ERJ Open Res.

[22] Stewart S., Fishbein M.C., Snell G.I. (2007). Revision of the 1996 working formulation for the standardization of nomenclature in the diagnosis of lung rejection. J Heart Lung Transpl.

[23] Folch E.E., Mahajan A.K., Oberg C.L. (2020). Standardized definitions of bleeding after transbronchial lung biopsy: a delphi consensus statement from the Nashville Working Group. Chest.

[24] Fruchter O., Fridel L., Rosengarten D. (2013). Transbronchial cryo-biopsy in lung transplantation patients: first report. Respirology.

[25] Yarmus L., Akulian J., Gilbert C. (2013). Cryoprobe transbronchial lung biopsy in patients after lung transplantation: a pilot safety study. Chest.

[26] Mohamed S., Mendogni P., Tosi D. (2020). Transbronchial cryobiopsies in lung allograft recipients for surveillance purposes: initial results. Transpl Proc.

[27] Sorbini M., Togliatto G., Mioli F. (2022). Validation of a simple, rapid, and cost-effective method for acute rejection monitoring in lung transplant recipients. Transpl Int.

